# Atrial Fibrillation Ablation During Hospitalization for Acute Heart Failure: Feasibility and Role of Pulsed Field Ablation

**DOI:** 10.1111/jce.16507

**Published:** 2024-11-26

**Authors:** Josef Marek, Predrag Stojadinović, Dan Wichterle, Petr Peichl, Jana Hašková, Eva Borišincová, Petr Štiavnický, Robert Čihák, Marek Šramko, Josef Kautzner

**Affiliations:** ^1^ Department of Cardiology Institute for Clinical and Experimental Medicine Prague Czechia; ^2^ 2nd Department of Medicine – Department of Cardiovascular Medicine Charles University Medical School I Prague Czechia

**Keywords:** acute heart failure, atrial fibrillation, catheter ablation, electroporation, posterior wall isolation, pulsed field ablation, thermal ablation

## Abstract

**Introduction:**

Atrial fibrillation (AF) can cause or aggravate heart failure (HF). Catheter ablation (CA) is an effective treatment for AF. This study focused on the feasibility and outcomes of emergent AF ablation performed during hospitalization for acute HF.

**Methods and Results:**

We retrospectively investigated patients who underwent emergent CA for AF during hospitalization for acute HF in 2018–2024. Arrhythmia recurrence was the primary endpoint. The combination of arrhythmia recurrence, HF hospitalization, and all‐cause death was the secondary endpoint. Patients were censored 1 year after the index procedure. We included 46 patients, 35% females, with median age of 67 [interquartile rage: 61, 72] years and left ventricular ejection fraction (LVEF) of 25 [23, 28]%. Thermal CA was performed in 14 patients, and pulsed field ablation (PFA) in 32 patients. Procedure time was significantly shorter with PFA compared to thermal CA (77 [57, 91] vs. 166 [142, 200] minutes, *p* < 0.001). Fluoroscopy time was longer with PFA (9.5 [7.6, 12.0] vs. 3.9 [2.9, 6.0] minutes, *p* < 0.001), with a borderline trend towards higher radiation dose (75 [53, 170] vs. 50 [30, 94] μGy.m^2^, *p* = 0.056). Extrapulmonary ablation was frequent (86% and 84% for thermal CA and PFA, *p* > 0.9). The estimated freedom from the primary endpoint was 79% after PFA and 64% after thermal CA (*p* = 0.44). The estimated freedom from the secondary endpoint was 76% after PFA and 57% after thermal CA (*p* = 0.43). LVEF improved by 24% ± 2% (*p* < 0.001) in patients with the first manifestation of HF and by 14% ± 4% (*p* = .004) in patients with decompensated HF diagnosed earlier.

**Conclusions:**

Emergent CA of AF during acute HF hospitalization is safe and associated with improved LVEF and good clinical outcomes. In the PFA era, the rate of these procedures is progressively increasing as they are readily available and easy to perform compared to thermal ablation.

**Abbreviations**
ACEI: angiotensin‐converting enzyme inhibitor
AF: atrial fibrillation
ARNI: angiotensin‐neprilysin inhibitor
AT: atrial tachycardia
BMI: body mass index
BNP: brain natriuretic peptide
BP: blood pressure
BSA: body surface area
CA: catheter ablation
CTI: cavotricuspid isthmus
HF: heart failure
HFr/mrEF: heart failure with reduced or mid‐range ejection fraction
ICE: intracardiac echocardiography
LA: left atrium
LVEF: left ventricular ejection fraction
PFA: pulsed field ablation
PV: pulmonary veins
PVI: pulmonary vein isolation
RFA: radiofrequency ablation
SGLT2: sodium‐glucose co‐transporter 2
TIA: transitory ischemic attack

## 1 Introduction

Atrial fibrillation (AF) and heart failure (HF) are among the most common cardiovascular conditions in the developed world, and their prevalence is steadily increasing [1, 2]. Moreover, they frequently coexist and perpetuate one another. AF is present in at least one‐fifth of patients with HF, and over half may have a history of AF [3, 4]. Not only is AF common in patients with HF, but it can by itself be a cause of acute HF or chronic HF decompensation [2, 3, 5].

Catheter ablation (CA) of AF has become the mainstay of treatment for AF and is more effective at maintaining sinus rhythm than medical therapy [1, 6]. Furthermore, in patients with HF, especially those with reduced left ventricular ejection fraction (LVEF), CA improves clinical outcomes, including mortality and hospitalization for HF decompensation [7, 8, 9].

Pulsed field ablation (PFA), a new alternative to thermal CA, has shown remarkable efficacy with shorter procedure durations, allowing for relatively fast substrate modification beyond pulmonary vein (PV) isolation (PVI) in patients with persistent AF [10, 11, 12, 13].

CA is usually performed as an elective procedure. In patients with acute HF caused by AF, electric cardioversion or amiodarone are often used for rhythm control [14]. However, when these therapies fail to achieve adequate rhythm control, emergent CA for AF during the hospitalization for acute HF offers a possibility for rhythm control both in the short and long term.

Accordingly, we evaluated patients hospitalized for acute HF who were ablated for AF during the hospitalization, comparing thermal CA to the PFA.

## 2 Methods

### 2.1 Population

We performed a retrospective cohort analysis of patients hospitalized for acute HF at our institution between 2018 and 2024 and had CA for AF performed during the initial hospital stay. Patients signed informed consent with the procedure. The study was approved by the local institutional review board and conforms to the Declaration of Helsinki.

Based on the ESC guidelines, HF was diagnosed with objective signs, elevated natriuretic peptides, and LVEF on echocardiography [2].

### 2.2 Acute Treatment

Based on institution standards, acute rhythm control of AF was performed using electrical cardioversion after left atrial (LA) thrombus was excluded using transesophageal echocardiography when clinically indicated. Amiodarone was used for acute pharmacological rhythm control [14]. CA was indicated based on the proposal of the attending physician and after consultation with the Heart Team at our institution.

### 2.3 Catheter Ablation Procedures

CA procedures were performed on uninterrupted anticoagulation. In patients undergoing thermal CA, mild conscious sedation was usually achieved with a combination of midazolam and fentanyl. Patients receiving PFA underwent either general anesthesia with desflurane or deep sedation with propofol. Unfractionated heparin was administered before the transseptal procedure, and ACT was maintained between 300 and 350 s. The transseptal puncture was guided by intracardiac echocardiography (ICE) imaging (AcuNav, Siemens Medical Solutions).

In patients undergoing radiofrequency ablation (RFA), LA was accessed using double transseptal puncture, and PVI was performed with the aid of a 3‐dimensional electroanatomic mapping system, CARTO 3 (Biosense Webster, USA), and ICE. A 3.5 mm irrigated‐tip catheter (NaviStar ThermoCool, Biosense Webster) was used for conventional point‐by‐point ablation. Energy settings were at the discretion of the operator, usually with a power of 20–30 W and ablation duration between 25 and 35 s, depending on the lesion location. Heparinized saline irrigation with a flow of 17 ml/min was used during the RF application. PVI was verified by a circular mapping catheter (Lasso, Biosense Webster).

One patient underwent cryoablation using the cryoballoon catheter (ArcticFront, Medtronic, USA) combined with RFA of the cavotricuspid isthmus (CTI) using the conventional 4 mm irrigated ablation catheter (ThermoCool, Biosense Webster).

PFA was performed using the Farapulse system (Boston Scientific, USA). PVs were isolated with a minimum of eight applications delivered per PV (four in basket configuration, four in flower configuration). Posterior wall ablation was done in the flower configuration with two applications per site and a 50% overlap between adjacent locations. Anterior wall lesions were created with three applications per site, starting from the anterior mitral annulus with a catheter in the basket configuration, then moving in a step‐wise fashion towards the right superior pulmonary vein with a simultaneous change of the catheter shape to flower configuration. Mitral isthmus ablation was done in a corridor from the mitral annulus to the left inferior pulmonary vein, both in basket and flower configuration, with four applications per site. Cavotricuspid isthmus ablation was done predominantly using the basket configuration, with three applications per location. All PF applications were done under direct ICE visualization to achieve adequate position and contact of the Farawave catheter with the targeted tissue.

Additional extrapulmonary ablation in RFA and PFA procedures were continued at the discretion of the operator. This was commenced especially when AF did not terminate or was still inducible after PVI. It usually started as electrogram‐guided ablation in LA to restore sinus rhythm or convert AF into atrial tachycardia (AT). In the case of PFA, anatomically‐guided ablation of the posterior LA wall was frequently attempted as the low‐risk empirical ablation step. All spontaneous or induced ATs were identified using entrainment and activation mapping and ablated.

### 2.4 Clinical Follow‐Up and Outcomes

Patients were followed according to institutional standards. This included a routine follow‐up visit 3 months after the procedure and then at 3–6 month intervals as clinically needed. ECG Holter monitoring was required at each clinical follow‐up visit. Antiarrhythmic treatment was based on individual clinical decisions.

The primary endpoint was AF/AT recurrence. The secondary endpoint was the combination of AF/AT recurrence, hospitalizations for HF, and all‐cause death. The study used no blanking period after ablation. Patients were censored 1 year after the index procedure.

### 2.5 Statistical Analysis

Continuous data were summarized using medians (interquartile range), and categorical data using percentages and proportions. Patients were divided into the thermal ablation cohort (RFA or cryoballoon ablation) and the PFA cohort. Student's *t*‐test, Mann‐Whitney U test, Chi‐squared test, and Fisher exact test were used to test for baseline differences, as appropriate.

Clinical outcomes were assessed using time‐to‐event analysis, with Kaplan‐Meier curves and log‐rank test used for group comparison. Changes in LVEF were modeled using linear mixed models for repeated measurements, and changes were reported as mean ± standard error.

A *p* < 0.05 was considered significant. R software version 3.5.1 (R Foundation for Statistical Computing, Vienna, Austria) was used for all analyses.

## 3 Results

### 3.1 Baseline Characteristics

A total of 46 patients out of 3491 (1.3%) ablated at our institution within the given period fulfilled the inclusion criteria (Table 1). No significant baseline differences between PFA and thermal CA were found except for the higher use of SGLT2 inhibitors in the PFA group. There was also a trend for higher use of angiotensin cascade blockers in the thermal CA group. Patients undergoing PFA were older and had higher BMI, but the differences were insignificant. All patients had reduced EF at admission except one, who had significantly increased natriuretic peptides and clinical signs of pulmonary edema. Of the 19 patients with known heart failure, the etiology was ischemic in 3 (16%), ischemic and valvular in 3 (16%), ischemic and dilated cardiomyopathy in one (5%), hypertrophic cardiomyopathy in one (5%) and dilated cardiomyopathy in 11 (58%), with no difference between PFA and thermal CA (*p* = 0.9) Admission LVEF in patients with known heart failure was 25 (20, 28)%, which was similar to patients without history of heart failure including those after heart transplant (25 [23, 28]%, *p* = 0.8). The last known LVEF before decompensation leading to hospitalization in patients with a history of heart failure was 39 (32, 43)%.

**Table 1:** Baseline characteristics.

Characteristic	Overall*, *N* = 46	PFA*, *N* = 32	Thermal CA*, *N* = 14	*p* value
Age (years)	67 (61, 72)	69 (61, 72)	64 (61, 69)	0.3
Female sex	16 (35%)	10 (31%)	6 (43%)	0.5
Height (cm)	176 (168, 182)	176 (169, 180)	178 (168, 184)	0.4
Weight (kg)	90 (78, 103)	91 (78, 101)	86 (79, 110)	0.8
BMI (kg/m^2^)	28.7 (26.7, 33.2)	29.7 (27.8, 33.4)	27.9 (25.2, 31.0)	0.8
Systolic BP (mmHg)	121 (108, 135)	120 (103, 134)	130 (122, 144)	0.10
Diastolic BP (mmHg)	80 (70, 89)	78 (70, 89)	80 (71, 87)	0.8
Admission NYHA class				0.089
II	5 (11%)	1 (3.1%)	4 (29%)	
III	19 (41%)	15 (47%)	4 (29%)	
IV	18 (39%)	13 (41%)	5 (36%)	
Intubated	4 (8.7%)	3 (9.4%)	1 (7.1%)	
Atrial fibrillation type				0.5
Paroxysmal	3 (6.5%)	2 (6.3%)	1 (7.1%)	
Persistent	21 (46%)	13 (41%)	8 (57%)	
First manifestation	22 (48%)	17 (53%)	5 (36%)	
Hospital admission rhythm				> 0.9
Atrial fibrillation	33 (72%)	23 (72%)	10 (71%)	
Sinus rhythm	7 (15%)	5 (16%)	2 (14%)	
Atrial tachycardia	6 (13%)	4 (13%)	2 (14%)	
Heart rate (s^−1^)	126 (98, 147)	122 (95, 152)	127 (102, 140)	0.9
History of heart failure				0.8
Yes	19 (41%)	14 (44%)	5 (36%)	
No	25 (54%)	17 (53%)	8 (57%)	
Heart transplant	2 (4.3%)	1 (3.1%)	1 (7.1%)	
Coronary artery disease	12 (26%)	10 (31%)	2 (14%)	0.3
Diabetes mellitus	14 (30%)	11 (34%)	3 (21%)	0.5
Stroke/TIA	4 (8.7%)	3 (9.4%)	1 (7.1%)	> 0.9
Peripheral Artery disease	3 (6.5%)	3 (9.4%)	0 (0%)	0.5
Arterial hypertension	26 (57%)	16 (50%)	10 (71%)	0.2
Dyslipidemia	24 (52%)	18 (56%)	6 (43%)	0.4
CHA_2_DS_2_‐VASc Score	3.0 (2.0, 4.0)	3.0 (2.0, 4.0)	3.0 (2.0, 3.0)	0.7
Chronic amiodarone	9 (20%)	6 (19%)	3 (21%)	> 0.9
Furosemide	19 (41%)	14 (44%)	5 (36%)	0.6
Aldosterone antagonists	14 (30%)	11 (34%)	3 (21%)	0.5
ACEI/sartan/ARNI	24 (52%)	14 (44%)	10 (71%)	0.084
Beta‐blockers	31 (67%)	20 (63%)	11 (79%)	0.3
SGLT2 inhibitors	9 (20%)	9 (28%)	0 (0%)	0.041
Digoxin	5 (11%)	5 (16%)	0 (0%)	0.3
Ejection fraction (%)	25 (23, 28)	28 (23, 28)	25 (23, 28)	0.6
LVDd (mm)	59 (54, 62)	59 (56, 63)	58 (53, 60)	0.4
LVDs (mm)	50 (43, 54)	50 (46, 54)	46 (41, 53)	0.4
Left atrial volume index (mL/m^2^)	52 (44, 60)	53 (46, 59)	49 (43, 58)	0.3
Left atrial diameter (mm)	48 (43, 51)	48 (43, 51)	48 (43, 50)	0.8
BNP (ng/L)	855 (472, 1841)	934 (505, 1886)	781 (229, 1473)	0.4
Creatinine (µmol/L)	114 (89, 154)	113 (86, 148)	115 (96, 150)	> 0.9

Abbreviations: ACEI, angiotensin‐converting enzyme inhibitor; ARNI, angiotensin‐neprilysin inhibitor; BMI, body mass index; BNP, brain natriuretic peptide; BP, blood pressure; CA, catheter ablation; LVDd, left ventricular diameter in diastole; LVDs, left ventricular diameter in systole; NYHA, New York Heart Association; PFA, pulsed field ablation; SGLT2, sodium glucose transporter 2; TIA, transitory ischemic attack.

* Median (IQR), *n* (%).

### 3.2 Management Before the CA

Urgent electrical cardioversion during admission was performed in 57% (8/14) of patients who later underwent thermal CA and 88% (28/32) who later underwent PFA (*p* = 0.047). The number of electrical cardioversions was 1 (1, 1) in the PFA group and 1 (0, 1) in the thermal CA group (*p* = 0.073); a maximum of 5 electrical cardioversions were performed. The duration from admission to first and last electrical cardioversion was 0 (0, 1) and 1 (0, 2) day, with no significant difference between PFA and thermal CA groups (*p* = 0.6 and 0.7, respectively).

Overall, 9 (20%) patients were on chronic amiodarone treatment with a dose of 200 (143, 200) mg, with no significant difference between PFA and thermal CA groups (*p* = 0.14). In 22 (48%) patients, the treatment with amiodarone was initiated during the index hospitalization, with a loading dose of 350 (75, 3200) mg at the time of the last electrical cardioversion and 2975 (1900, 4475) mg at ablation, with no significant differences between the PFA and thermal CA groups (*p* > 0.9 and *p* = 0.2, respectively). In these patients, only intravenous amiodarone up to ablation was given in 6 (27%) patients, combined intravenous and oral loading in 12 (55%) patients, and oral loading in 4 (18%) patients, with no differences between PFA and thermal CA groups (*p* > 0.9).

CA was indicated either in case of in‐hospital recurrence of AF/AT in 28 patients, of whom 23 were already on amiodarone treatment, or in case of perceived high risk for recurrence in 18 patients.

The duration from admission to CA was 6 (6, 7) days in the thermal CA group and 7 (2, 8) days in the PFA group (*p* = 0.8). The duration from the last electrical cardioversion to ablation was 6 (3, 7) days in the thermal CA group and 3 (1, 7) days in the PFA group (*p* = 0.5).

CA was performed in sinus rhythm in 64% (9/14) and 63% (20/32) of patients in the thermal CA and PFA groups, respectively (*p* > 0.9).

### 3.3 Procedural Characteristics

Overall, the number of eligible cases progressively increased over time, predominantly after switching from thermal ablation to PFA between 2021 and 2022 (Central Illustration A). The last thermal ablation was performed in October 2021, and the first PFA was performed in March 2022.

**Central Illustration:**
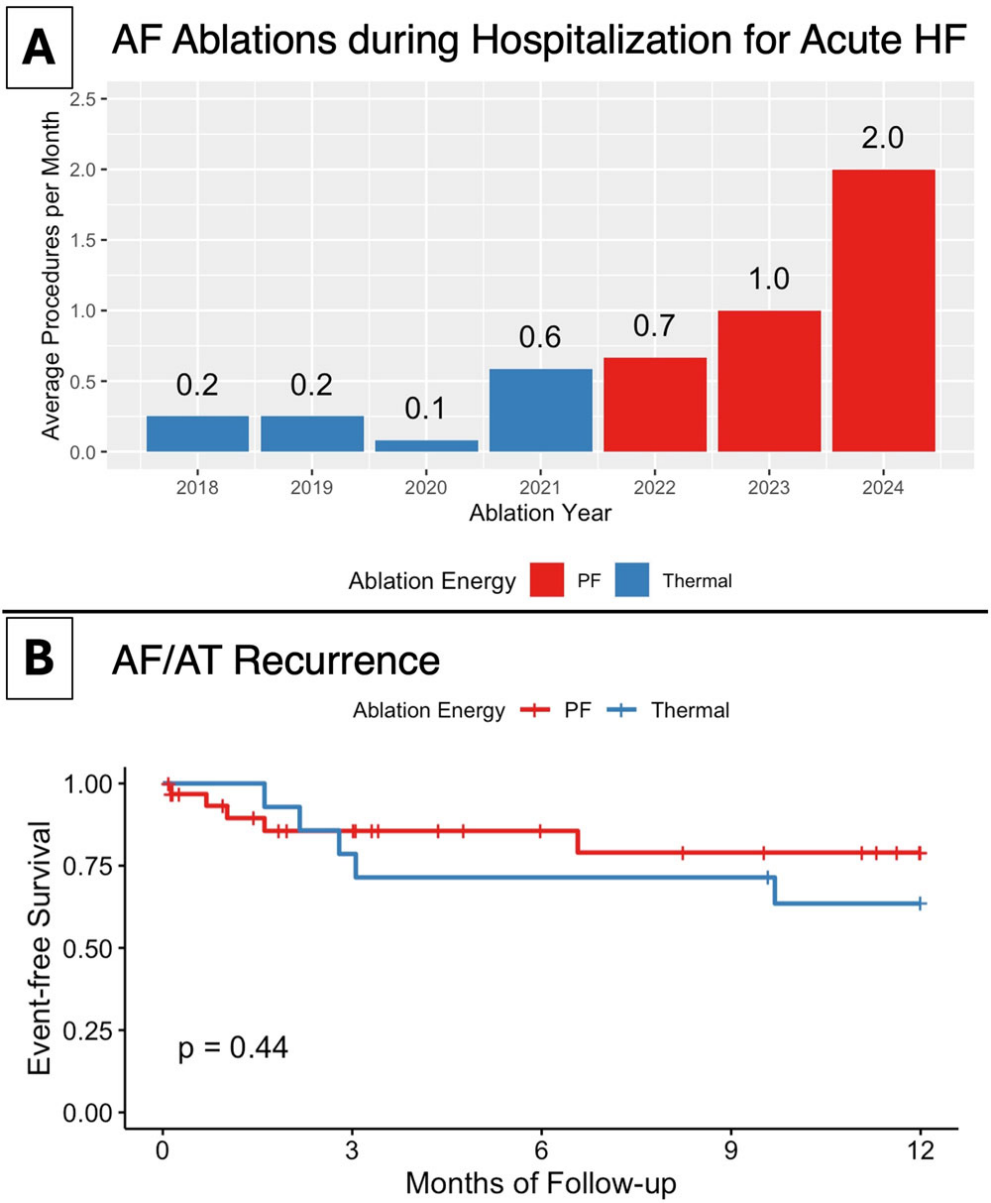
(A) Atrial fibrillation (AF) ablations during hospitalization for acute heart failure. The plot shows an increasing trend in acute AF ablations. The columns represent the number of ablations per month in the particular year in categories by ablation energy (pulsed field (PF) ablation energy in red, thermal energy in blue). (B) Freedom from AF/AT recurrence. The Kaplan‐Meier curves for the primary endpoint are categorized by ablation energy (PF ablation energy in red, thermal energy in blue). AT, atrial tachycardia; HF, heart failure.

**Figure 1:**
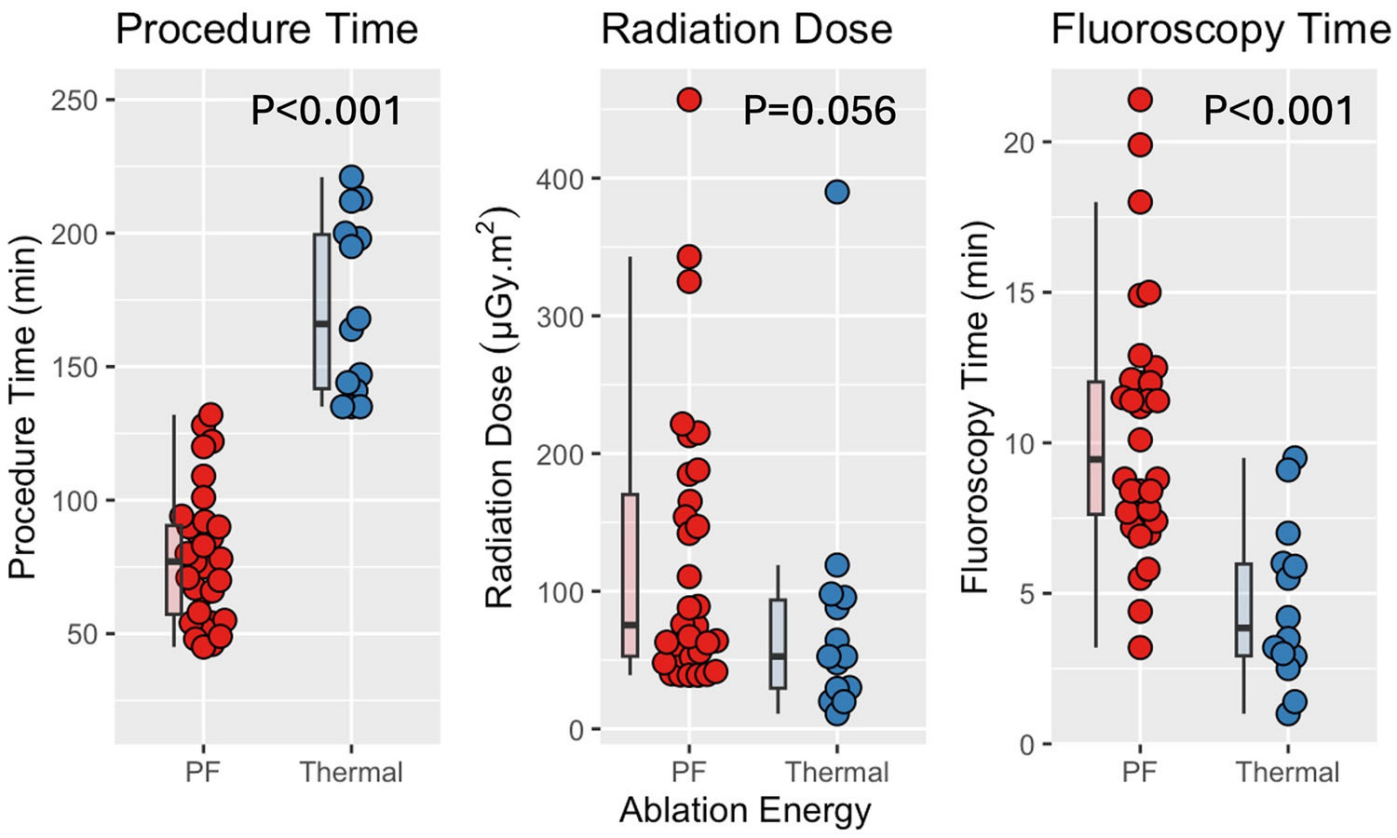
Procedural characteristics. PF procedures (in red) are compared to thermal ablation procedures (in blue). PF, pulsed field.

Procedure duration, fluoroscopy time, and radiation dose are shown in Figure 1. PFA was associated with significantly shorter procedure durations, with duration decreasing from 166 (142, 200) to 77 (57, 91) minutes (*p* < 0.001). Fluoroscopy time was significantly longer in PFA (9.5 [7.6, 12.0] vs. 3.9 [2.9, 6.0] minutes, *p* < 0.001), and a borderline trend towards higher radiation dose for PFA was observed (75 [53, 170] vs. 50 [30, 94] μGy.m^2^, *p* = 0.056).

**Figure 2:**
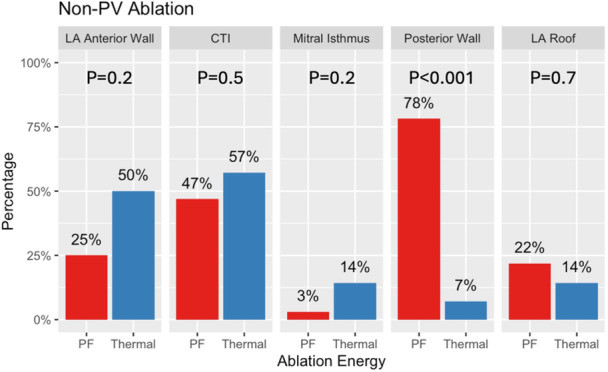
Non‐PV ablation sites. The differences in non‐PV ablation according to ablation energy are shown (PF energy in red, thermal energy in blue). Anterior wall ablation was more common in thermal ablation and posterior wall ablation in PF ablation. CTI, cavotricuspid isthmus; LA, left atrium; PV, pulmonary veins; PF, pulsed field.

PVI was achieved in all patients. Additional ablation lesions beyond PVI were common (overall 85% [39/46]; 86% [12/14] and 84% [27/32] for thermal CA and PFA, *p* > 0.9) and are summarized in Figure 2. Posterior wall ablation was more common in the PFA group (78% [25/32] vs. 7% [1/14], *p* < 0.001). Anterior wall ablation was numerically more common in thermal CA, but the difference was not statistically significant (50% [7/14] vs. 25% [8/32], *p* = 0.2). The reason for anterior wall ablation was perimitral flutter in four patients, complex fractionated atrial electrograms during arrhythmia in eight patients (four in AF, four in AT), focal AT from the anterior wall in two patients, and extensive scarring during sinus rhythm in one patient. Mitral isthmus ablation was performed in one thermal CA and one PFA patient to treat perimitral flutter. It was unsuccessfully attempted in another thermal CA patient in whom anterior wall ablation was subsequently performed.

Several ATs were noted during the procedures. Perimitral atrial flutter was induced in four patients and developed spontaneously in two patients. It was treated by anterior wall ablation in three, mitral isthmus ablation in two and anterior wall ablation after unsuccessful mitral isthmus ablation in one patient. Typical atrial flutter was present in four patients at the onset of the procedure, was induced in seven patients, and developed spontaneously in four patients; all were treated by cavotricuspid isthmus ablation. Several microreentrant ATs were observed: ongoing AT in one patient, which was ablated at the anterior wall; induced ATs in six patients (three ablated at the anterior wall, one at the ostium of LA appendage and two right‐atrial ATs ‐ one terminated spontaneously and the other ablated at the inferolateral right atrium); and spontaneously developed ATs in five patients (two ablated at the anterior wall, one septal terminated spontaneously, 1 ablated at the mitral annulus and one ablated at posterior tricuspid annulus). All patients ended the CA procedure in sinus rhythm.

In one patient in the PFA group, in whom the procedure started in deep sedation, intubation and conversion to general anesthesia were necessary because of hypoventilation and the need for airway management. Despite this, CA was completed successfully without adverse sequelae. No other complications or adverse effects were observed in the entire cohort.

### 3.4 Hospital Discharge

All patients, except one in the PFA group, were in sinus rhythm at discharge. There was no significant difference in time from ablation to discharge between PFA (2.5 [2.0, 4.0]) and thermal CA (3.0 [2.0, 6.3], *p* = 0.6). At discharge, 50% (7/14) of patients in the thermal CA group and 66% (21/32) in the PFA group were prescribed amiodarone (*p* = 0.3). Of these patients, 18 (64%) received 200 mg daily. Further 6 (21%) received 400 or 600 mg as a part of the loading dose, with a reduction to 200 mg after 6 g of amiodarone loading; one of them received amiodarone at discharge for ventricular premature contractions. Four patients (14%) received a reduced dose of 143 or 114 mg per day (given as 200 mg 4–5 times a week). Four patients received amiodarone acutely before ablation but were discharged without antiarrhythmic treatment in sinus rhythm. None of these 4 patients had arrhythmia recurrence. No significant difference between PFA and thermal CA in amiodarone dosage was noted (*p* = 0.2).

One patient in the thermal CA group with restored normal LVEF received propafenone.

### 3.5 Clinical Outcomes

Patients were followed up for 277 (95, 365) days in the PFA group and 365 (365, 365) in the thermal CA group.

There were five arrhythmia recurrences and one reablation in the PFA group, and five arrhythmia recurrences and two reablations in the thermal CA group. Estimated freedom from the primary endpoint at 12 months was 79% in the PFA group and 64% in the thermal CA group (Central Illustration B, *p* = 0.44).

At the redo procedure in the PFA patient, reconnection of the common trunk of left PVs, posterior wall, and CTI was noted. In one thermal CA patient with pulmonary vein isolation only, a gap was noted in the right superior pulmonary vein. In the other thermal CA patient with pulmonary isolation combined with roof and anterior lines, no gaps in veins or lines were observed. After reablation, none of them had arrhythmia recurrence during the 1‐year follow‐up.

Two and three patients were hospitalized for HF in the PFA and thermal CA groups, respectively. In two patients from the thermal CA group and one patient from the PFA group, AF recurrence was a contributing cause of HF hospitalization. At the last follow‐up, 37 (80%) patients had angiotensin pathway inhibitors, 40 (87%) beta‐blockers, and 29 (63%) mineralocorticoid receptor antagonists, with no difference between PFA and thermal CA (NS for all). In the PFA group, significantly more patients were prescribed SGLT2 inhibitors (23 patients, 72%) than in the thermal CA group (1 patient, 7%, *p* < 0.001).

**Figure 3:**
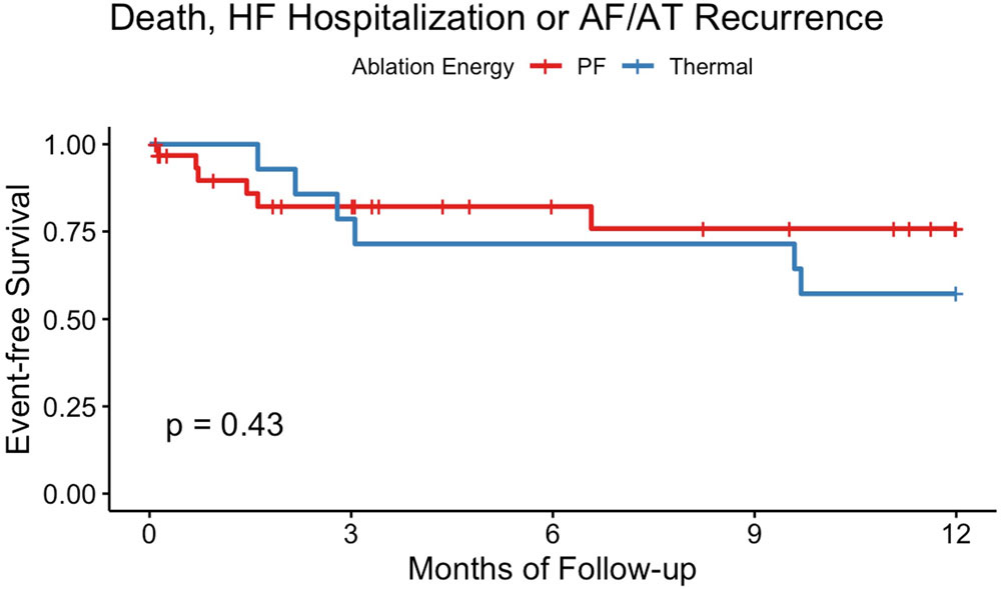
Freedom from all‐cause death, HF hospitalization or AF/AT recurrence. The Kaplan‐Meier curves for the secondary endpoint are categorized by ablation energy (PF energy in red, thermal energy in blue). AF, atrial fibrillation; AT, atrial tachycardia; HF, heart failure; PF, pulsed field.

Two patients died, one in each study group. None of the deaths were related to the procedure. Estimated freedom from the secondary endpoint at 12 months was 76% in the PFA group and 57% in the thermal CA group (Figure 3, *p* = 0.43)

No significant differences in the continuation of antiarrhythmic therapy were seen between the study groups. Of patients without AT/AF recurrence, 29% versus 46% were on antiarrhythmic therapy at 6 months and 6% versus 27% at 12 months in the PFA and thermal CA groups, respectively (*p* = 0.3). The last daily dose of amiodarone before discontinuation or end of follow‐up was 200 (143, 200) mg with PFA and 143 (114, 143) mg in thermal CA (*p* = 0.089). Although there was a trend towards better survival without AF/AT in patients discharged on amiodarone (84% ± 7% at 1 year) than those without (49% ± 16%), it did not reach statistical significance (*p* = 0.12). All the patients remained in rhythm control strategy during the follow‐up.

### 3.6 Follow‐Up Echocardiography

**Figure 4:**
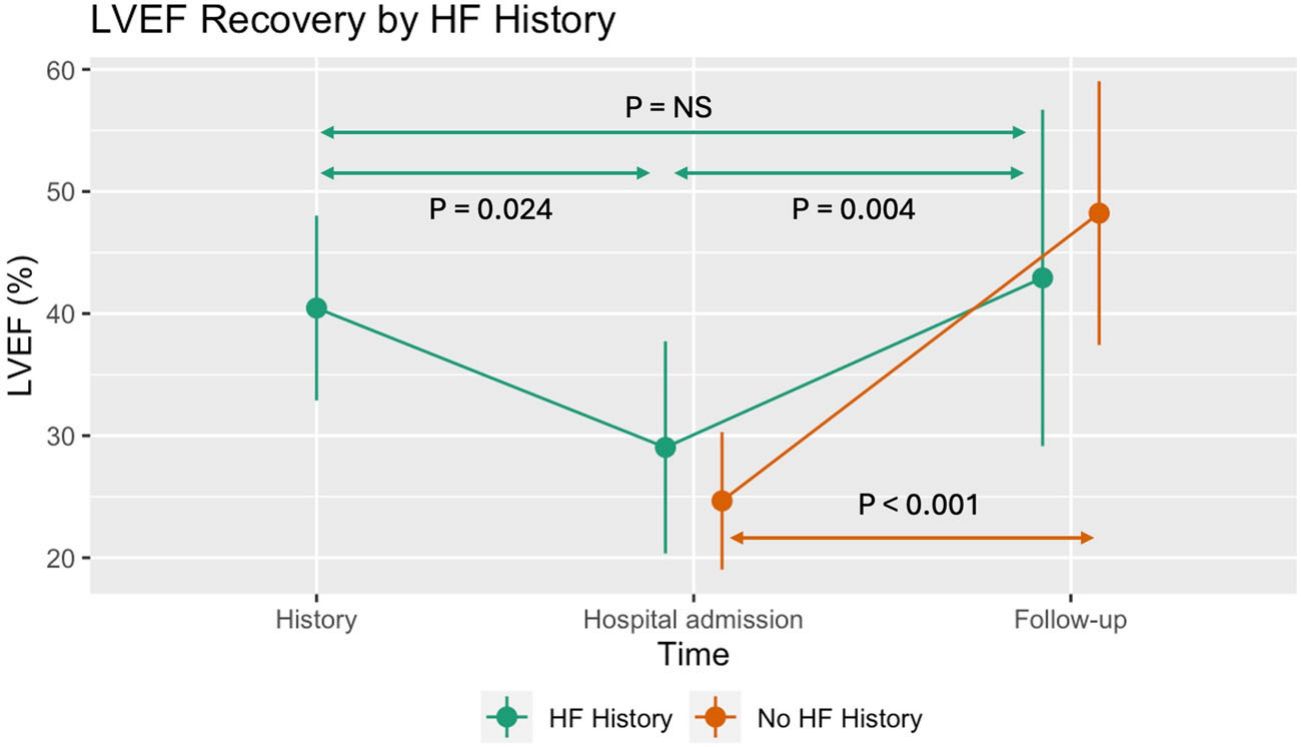
Changes in LVEF after AF ablation. Line plots for temporal change of LVEF categorized by HF history (present in green, absent in orange). Error bars denote the crude standard deviations. HF, heart failure; LVEF, left ventricular ejection fraction.

Follow‐up echocardiography was available in 35 patients 98 (69, 167) days after CA. As shown in Figure 4, there was a significant improvement in LVEF after CA both in patients who had the first manifestation of HF (+24% ± 2%, *p* < 0.001) and in patients presenting with decompensated HF (+14% ± 4%, *p* = 0.004). The improvement was significantly better in the former group (difference of +10% ± 4%, *p* = 0.027 for interaction). In patients with a history of HF, LVEF was significantly reduced at admission compared to chronic values (−12% ± 4%, *p* = 0.024). There were no significant differences in LVEF improvement between the PFA and thermal CA groups. Although patients with AF/AT recurrence had numerically a smaller LVEF improvement compared to the rest of the cohort, it did not reach statistical significance (−4% ± 5%, *p* = 0.4).

## 4 Discussion

To the best of our knowledge, this is the first study demonstrating that emergent CA for AF during the index hospitalization for acute decompensated HF is feasible in selected patients and is associated with good clinical outcomes. Furthermore, the study showed that introducing PFA resulted in a progressive increase of acutely indicated AF ablation procedures at our institution, as these can be performed faster (even in cases requiring extensive ablation) and are presumably safer than thermal CA.

### 4.1 Atrial Fibrillation Treatment in Heart Failure

Currently, the initial approach in patients with AF in acute HF is rate control using amiodarone and possibly digoxin [14, 15]. However, rhythm control can be considered, and in hemodynamically unstable patients, electrical cardioversion is preferable [1, 14]. This was often the case in our population, where AF with fast ventricular response necessitated early electrical cardioversion in most patients. Unfortunately, long‐term rhythm control after cardioversion can be challenging in such a population, with frequent in‐hospital recurrences seen in our group. In older studies using antiarrhythmic drugs, rhythm control was not superior to rate control in the general population as well as patients with HF [16, 17]. But more recently, early rhythm control has been shown to improve outcomes in the general population of AF as well as patients with HF in randomized controlled trial settings [18, 19]. Nevertheless, arrhythmia recurrence is not uncommon, even when using amiodarone to maintain sinus rhythm [8, 20]. This was also seen in our study, where most recurrences during hospitalization were already on amiodarone treatment. Furthermore, amiodarone is associated with significant extracardiac toxicity [21].

### 4.2 Catheter Ablation

CA is more effective at maintaining sinus rhythm compared to antiarrhythmic therapy [6]. In patients with HF and reduced LVEF, CA results in a better long‐term prognosis [7, 8]. A subanalysis of the CABANA trial has also shown significant benefit in the subgroup of patients with HF, who had mostly preserved LVEF [22]. Recently, the CASTLE‐HTx trial has demonstrated the effect of CA in patients with advanced HF. In this group, CA has shown a significant improvement in the composite endpoint of death, LVAD implantation, or urgent heart transplant [9]. Importantly, CA was not associated with an increased complication rate, which is in agreement with the current study.

### 4.3 Timing of Catheter Ablation

CA is also an effective first‐line AF treatment strategy [23, 24]. Furthermore, multiple observational studies have associated better outcomes with earlier rhythm control treatment, and a shorter time from diagnosis to CA is also associated with improved outcomes [25, 26, 27].

In patients with HF, an observational study of the Japanese registry of HF has compared patients with acute HF and AF who underwent CA within 90 days after admission for HF to those who did not. Patients with early CA had lower all‐cause, cardiovascular, and HF mortality in both unmatched and matched analyses [28]. In our study, we achieved a short waiting time from the initial presentation to ablation, with a median of 6–7 days.

### 4.4 Pulsed Field Ablation

PFA is a promising technology that allows easier and faster AF ablation than thermal CA technologies [12]. PVI can be achieved quickly and reliably, and this technology is safe for extracardiac tissue, especially the phrenic nerve and esophagus [11, 13]. In patients with persistent AF, PFA can also be used for ablation beyond PVs, especially targeting the posterior wall [10, 29, 30]. In patients with HF and persistent AF, extrapulmonary ablation appears to be a reasonable strategy, and PFA is able to achieve this with acceptable procedure duration.

In our data set, the median procedure time for thermal CA was 166 min compared to the mean duration of 121 min in the control arm (thermal PVI for paroxysmal AF) of the ADVENT trial [12]. It reflects more complex ablation in patients with persistent AF in the setting of HF. The median duration of our PFA procedures was 77 min, which was significantly shorter than the duration of our thermal CA. It was also shorter compared to the PFA arm of ADVENT (mean of 105 min), and the same as the median time of 77 min in patients with HF and reduced or mid‐range LVEF (HFr/mrEF) from the MANIFEST‐PF registry [13]. PFA duration also compares favorably to the average thermal CA duration in CASTLE‐HTx, which was 95 min. The effectiveness of PFA was comparable to previous trials, with freedom from arrhythmia at 12 months in 79% of our PFA patients. We did not use the blanking period because we believe that arrhythmia recurrence, even in the early period, can be detrimental to patients with HF. Our results compare favorably to the ADVENT trial with 73.3% and 71.3% success rates for PFA and thermal CA, respectively. They are also numerically better than the 67.5% success rate in patients with HFr/mrEF from the MANIFEST‐PF heart failure substudy [31]. However, comparison of effectiveness is difficult due to a highly selected population in our study.

### 4.5 Ablation Beyond Pulmonary Veins

Non‐PV lesions were created in 85% of patients in our study. The most common additional lesions were the anterior wall in thermal CA and the posterior wall in PFA. This is consistent with some previous studies in HF. In the AATAC trial, posterior wall isolation was performed in 78% of the patients [8]. In CASTLE‐AF, additional lesions were performed in 51% (77/151) of the patients, with the most common lesions being roof line (39/151), right atrial isthmus (29/151), and left atrial isthmus (26/151). Other studies have reported lower percentages of non‐PVI lesions. In CASTLE‐HTx, additional lesions were created in 37% of patients in the ablation arm. The most common additional lesions were the anterior line (19%) and right atrial ablation (19%). In the MANIFEST‐PF HF substudy, 33% of patients in the HFrEF/mrEF group had additional lesions. However, the posterior wall was also the most common lesion created [31].

### 4.6 Left Ventricular Function After Ablation

We have observed significant improvements in LVEF after CA. In CASTLE‐HTx, CA resulted in an average LVEF increase of 6.7% and 7.8% points at 6 and 12 months of follow‐up, respectively. The difference in nonischemic HF in the CAMERA‐MRI trial was even more pronounced, with an 18.3% points improvement [32]. Improvement was most striking in patients without LGE, with up to 22.3% points. On the other hand, no significant improvement has been shown in the AMICA trial [33]. In our study, we have seen significant improvement in patients with a history of HF comparable to the CAMERA‐MRI trial and even higher in patients without a history of HF. These considerable improvements must be interpreted in the clinical context since patients had acute HF with rapid ventricular rates at admission, contributing to the LVEF reduction. Furthermore, the observed improvement is a joint effect of CA for AF and the optimization of HF medical therapy [2]. The adherence to heart failure medication at 1 year was high, with only a significant difference between thermal CA and PFA in the SGLT2 inhibitor usage, which can explained by the fact that SGLT2 inhibitors were not routinely available in 2018‐2021 when thermal CA was performed.

Nevertheless, seeing these considerable improvements after intensive combined therapy for both conditions is reassuring.

### 4.7 Study Limitations

Our study has several limitations. First, it is an observational retrospective study on a highly selected population with inherent biases. Whether this therapeutic strategy is superior to postponed elective ablation would need to be shown in a randomized controlled trial. Such trial should also evaluate the cost‐effectiveness of each approach. Also, comparing the thermal CA and PFA groups is limited by a small sample size, a different period when CA and PFA were performed, and a non‐randomized design.

Extended ECG monitoring was not systematically available. However, the most important endpoint for this population is clinical benefit: AF recurrence and HF hospitalization captured during routine clinical follow‐up performed at our institution.

Additional ablation lesions were placed at the discretion of the operator. While PFA allows safe posterior wall isolation, no data has conclusively proven that this approach is superior to PVI‐only procedures or to other extrapulmonary (including anterior wall) lesions [29, 30, 34]. However, most of our patients had, per definition, AF that was at least acutely difficult to manage using cardioversion and pharmacological therapy. Left atrial dilation was also significant. Both these facts (not to mention the HF itself) point to more advanced atrial disease. Despite the conflicting results of additional ablation, PVI alone might not be enough for these patients [34, 35]. In the case of PFA, a randomized clinical trial (NCT05922917–The PIVCO trial) is ongoing to answer this question.

## 5 Conclusion

CA of AF during the index hospitalization for AF‐related acute HF is feasible, safe and results in good clinical outcomes, including significant improvements in LVEF. Pulsed field technology has significantly shortened the procedure duration compared to thermal CA and made it readily available and easy to perform. It has lowered the bar for choosing this intervention as an emergent treatment for AF in the setting of acute or exacerbated HF.

## Author Contributions

Substantial contributions to the conception and design or the acquisition, analysis, or interpretation of the data: J.M., P.S., P.P., J.H., E.B., P.Š., R.Č., M.Š., J.K. Substantial contributions to the drafting of the articles or critical revision for important intellectual content: J.M., P.S., P.P., J.H., E.B., P.Š., R.Č., M.Š., J.K. Final approval of the version to be published: J.M., P.S., P.P., J.H., E.B., P.Š., R.Č., M.Š., J.K. Agreement to be accountable for all aspects of the work in ensuring that questions related to the accuracy or integrity of any part of the article are appropriately investigated and resolved: J.M., P.S., P.P., J.H., E.B., P.Š., R.Č., M.Š., J.K.

## Disclosure

Josef Kautzner reports personal fees from Biosense Webster, Boston Scientific, GE Healthcare, Medtronic, and St. Jude Medical (Abbott) for participation in scientific advisory boards, and has received speaker honoraria from Biosense Webster, Biotronik, Boston Scientific, Medtronic, ProMed CS, St. Jude Medical (Abbott) and Viatris. Petr Peichl has received speaker honoraria from St Jude Medical (Abbott) and has served as a consultant for Biotronik and Boston Scientific. The remaining authors have no disclosures.

## Data Availability

The data that support the findings of this study are available from the corresponding author upon reasonable request.
